# Soil Nematode Responses to Increases in Nitrogen Deposition and Precipitation in a Temperate Forest

**DOI:** 10.1371/journal.pone.0082468

**Published:** 2013-12-06

**Authors:** Xiaoming Sun, Xiaoke Zhang, Shixiu Zhang, Guanhua Dai, Shijie Han, Wenju Liang

**Affiliations:** 1 State Key Laboratory of Forest and Soil Ecology, Institute of Applied Ecology, Chinese Academy of Sciences, Shenyang, China; 2 Northeast Institute of Geography and Agroecology, Chinese Academy of Sciences, Changchun, China; 3 Research Station of Changbai Mountain Forest Ecosystems, Chinese Academy of Sciences, Erdaobaihe, China; 4 College of Resources and Environment, University of Chinese Academy of Sciences, Beijing, China; DOE Pacific Northwest National Laboratory, United States of America

## Abstract

The environmental changes arising from nitrogen (N) deposition and precipitation influence soil ecological processes in forest ecosystems. However, the corresponding effects of environmental changes on soil biota are poorly known. Soil nematodes are the important bioindicator of soil environmental change, and their responses play a key role in the feedbacks of terrestrial ecosystems to climate change. Therefore, to explore the responsive mechanisms of soil biota to N deposition and precipitation, soil nematode communities were studied after 3 years of environmental changes by water and/or N addition in a temperate forest of Changbai Mountain, Northeast China. The results showed that water combined with N addition treatment decreased the total nematode abundance in the organic horizon (O), while the opposite trend was found in the mineral horizon (A). Significant reductions in the abundances of fungivores, plant-parasites and omnivores-predators were also found in the water combined with N addition treatment. The significant effect of water interacted with N on the total nematode abundance and trophic groups indicated that the impacts of N on soil nematode communities were mediated by water availability. The synergistic effect of precipitation and N deposition on soil nematode communities was stronger than each effect alone. Structural equation modeling suggested water and N additions had direct effects on soil nematode communities. The feedback of soil nematodes to water and nitrogen addition was highly sensitive and our results indicate that minimal variations in soil properties such as those caused by climate changes can lead to severe changes in soil nematode communities.

## Introduction

Climate changes due to anthropogenic impacts directly or indirectly affect ecosystem structure and functioning [1]. The ecological consequences arising from nitrogen (N) deposition are becoming increasingly important for climate change studies in the world [2,3]. China is experiencing intense air pollution caused largely by anthropogenic emissions of nitrogen [4]. These emissions result in the deposition of atmospheric nitrogen in terrestrial ecosystems, with implications for greenhouse gas balances and biological diversity. Nitrogen deposition is predicted to increase in the coming decades in China [5]. Especially, more atmospheric nitrogen depositions due to anthropogenic activities occur in forest ecosystems, which cause diverse effects on the constitution and function of forest ecosystems and can even lead to degradation of forests [6]. Atmospheric N deposition in forest ecosystems can affect the leaching or retaining capacity and availability of soil N, and reduce soil pH [7]. Furthermore, nitrogen deposition can alter the nutrient distribution between soil organisms and plants through affecting soil moisture, pH, C/N and N availability, and influencing the growth, activity and community composition of soil biota [8]. However, little information on the effects of N deposition on soil biota communities is available, especially in the temperate forests of Northeast China, which limits our understanding of how N additions influence the processes and functioning of forest soil ecosystems.

Precipitation is another crucial factor influencing soil biota communities in forest ecosystems. Compared with the increasing atmospheric N deposition, changes in precipitation will have a more direct impact on soil organisms [9]. Moreover, the effects of N deposition on soil ecosystems may vary depending on the changes in precipitation [10] as soil N cycling processes, and N pool size and flow are closely related to precipitation [11–13]. The relationship between N cycle rate and soil biota community composition relies on the quantity and intensity of precipitation [14,15]. Synergistic effects of precipitation and N deposition on soil biota communities will be probably more complex.

Soil biota responses to N deposition and precipitation indicate the feedbacks of terrestrial ecosystems to climate change [3]. As one of the most important soil biota, soil nematodes are widespread and highly diverse, occupying multiple trophic positions in the soil food web and nematodes are increasingly used as an indicator of soil environmental change [16–18]. Soil nematodes play a crucial role in the terrestrial N cycle by accelerating the release of ammonium from microorganisms (bacteria and fungi). Free-living nematodes that feed on bacteria and fungi contribute as much as 27% of the readily available nitrogen in the soil [19]. Previous studies have documented the responses of nematode community composition and diversity to nitrogen addition. Van der Wal et al. (2009), for example, reported that the diversity of soil nematodes increased with increasing plant productivity after nitrogen addition [20]. However, another study showed that N addition decreased total nematode abundance and diversity, but that responses also varied among trophic groups [18]. The numbers of opportunistic bacterivores increased after N addition in forest ecosystems [21]. Nitrogen addition can affect soil nematode communities through changing the patterns of belowground C allocation to soil biota in forest ecosystems [22,23]. It must also be noted that responses of soil nematodes to N addition often vary with application time, soil type, sampling depth, and ecosystem type. Changes in precipitation also influence soil nematode abundance and community composition because nematodes depend upon water for movement and to migrate towards their prey [24]. Landesman et al. (2011) reported that total nematode abundance increased with increasing precipitation and that bacterivores were most sensitive to drought [15].

Climatic changes will not happen in isolation [27], and several studies have shown that soil biota respond differently to various climate change factors [25,26]. Interestingly, a number of studies have shown that the effects of multiple climate change factors on soil biota are interactive, rather than additive [8,22,28,29]. Kardol et al. (2011), for example, demonstrated that the single and combined effects of elevated CO_2_, warming, and change in precipitation regime shaped the abundance and community structure of soil microarthropods. Li et al. (2009) found there were significant interactions between N fertilization and elevated CO_2_ on how they affected the total nematode abundance and the abundance of different trophic groups [30]. So far, although the combined effects of elevated CO_2_, temperature and precipitation on soil biota communities has been reported in some ecological researches, only a few studies have investigated the effects of both precipitation and nitrogen addition on soil biota communities. In particular, studies on the interaction of precipitation and N deposition on soil nematode communities are largely lacking. Therefore, the objectives of this study were to determine the effects of water, nitrogen addition and their interactive effects on soil nematode community composition, and to explore the underlying mechanisms of soil nematode response to N deposition and precipitation in temperate forests.

## Materials and Methods

No specific permits were required for the described field studies and activities. The location is not privately owned or protected in any way and the field studies did not involve endangered or protected species.

### Study site

The study was conducted in the Changbai Mountain Natural Reserve (41°23′–42°36′N, 126°55′–129°00′E), Jilin Province, Northeast China. This area belongs to a typical temperate continental monsoon climate. The study plot was established in an old growth forest (200 years old) of broad-leaved Korean pine mixed forest (42°24′N, 128°06′E) with 760 m a.s.l. in Changbai Mountain. In the plot, the annual mean temperature is 3.5 °C, and the annual mean precipitation is 700 mm, 70–80% of which falls during the growing season from May to October [31–33]. The soil, developed from volcanic ash, is classified as Eutric cambisol (FAO classification). Tree species are dominated by a mixture of coniferous *Pinus koraienssis*, *Fraxinus mandschurica*, *Acer mono*, *and Tilia amurensis* [34,35].

### Experimental design and sampling

The experiment with a split-plot design was established in September 2009, where the water addition treatment (no addition or 30% addition) was applied to main plots and N addition (no addition or 50 kg N ha^-1^year^-1^) in sub-plots. The four treatments were control (no water and nitrogen addition), N+ (N addition with 50 kg N ha^-1^year^-1^), W+ (30% water addition), W+N+ (30% water and 50 kg N ha^-1^year^-1^addition). There were three replicates of each treatment. A total of twelve 50 m ×50 m plots with a buffer zone of > 20 m between any two plots were randomly established. NH_4_NO_3_ was diluted in 40 L of deionized water. In each N addition area, the application was done monthly (six times from May to October in every year) with a sprayer. The application rates of 50 kg N ha^-1^year^-1^ were about double the annual total N deposition (23 kg N ha^-1^year^-1^) in this area [36]. To collect natural rainfall to be used in additional water treatment, six extra plots (one for each W+ plot) were set up that were covered for 30% with clear PVC panels and all the natural rainfall was collected and diverted into collection tanks. This natural rainfall was sprinkled to rainfall addition plots (W+ and W+N+ treatments) through a pump connected to a hose. 

Soil samples from all plots were taken on August 10, 2012 after applying the treatments for almost 3 years. After the removal of aboveground plant debris, soil samples were extracted using a soil corer (5 cm diameter) and separated into organic (O) and mineral (A) horizons in the field [37]. A total of 5 cores were collected and mixed together to form a composite sample per subplot as one replicate. In our study site, the O horizon is approximately at 0-10cm depth with clay loam, and the A horizon at 10-25 cm depth with sandy clay loam. The moist samples were stored at 4°C until analysis.

### Soil analysis

Soil moisture (SM) was measured gravimetrically. The value of pH was measured in a 1:2.5 soil/water solution. The total soil organic carbon (SOC) was determined by potassium dichromate oxidation, which was assumed to be equal to the total C because the soil was free of carbonate [38]. The Kjeldahl method was used to determine the soil total N (TN) of samples [39]. The concentrations of nitrate N (NO_3_^-^-N) and ammonium N (NH_4_^+^-N) in the filtrates were determined using a flow injection auto analyzer (AutoAnalyzer3, Digital Colorimeter). Soil microbial biomass carbon (MBC) and nitrogen (MBN) were extracted using the fumigation-extraction method and the corresponding soil extracts was measured using a TOC analyser (Multi C/N 3000, Analytik Jena, Germany). Both MBC and MBN were calculated as the differences in ﬁltrates between the fumigated and unfumigated soil. The *K*_*C*_ and *K*_*N*_ factors for MBC and MBN were 0.38 and 0.54, respectively [40,41]. 

### Nematode community analysis

A total of 100 g fresh soil taken from the composite sample was used to extract nematodes with a modified cotton-wool filter method [42]. After counting the total number of nematodes, 100 specimens per sample were randomly selected and identified to genus level using an inverted compound microscope. If the total number of nematodes in the sample was less than 100, all the nematodes were identified. Nematode populations were expressed as number of nematodes per 100 g dry soil according to the soil moisture. The nematodes were assigned to the following trophic groups characterized by feeding habits (1) bacterivores (BF); (2) fungivores (FF); (3) omnivores-predators (OP) and (4) plant-parasites (PP) following Yeates et al. (1993) [43]. The nematode genus *Filenchus* was allocated to the fungivores in this study following recent evidence that has shown that this group feeds on fungi rather than on plant roots [44–46]. 

The following nematode ecological indices were calculated: trophic diversity (TD), Simpson's index for dominance (λ), the Shannon-Weaver diversity index (H'), maturity index (MI), the nematode channel ratio (NCR), and channel index (CI) [47–50].

### Statistical analysis

Nematode abundances were ln (x+1) transformed prior to the statistical analysis for normality of the data. The general linear model (GLM) procedure with multivariate analyses of variance (MANOVA) for a split-plot design were performed to test the effects of N addition, water addition and their interactions on soil properties and nematodes. All statistical analyses were performed using SPSS (vers.13.0; SPSS, Chicago, IL, USA).

Structural equation modeling (SEM) was used to gain a mechanistic understanding of how water and N addition influenced soil nematode communities. SEM is a multivariate statistical method that allows for hypothesis testing of complex path-relation networks [51]. We constructed an *a priori* model based on a literature review and our knowledge of the relative contributions and interactions of the following predicators. Soil properties and nematode communities were treated as latent variables. In the initial model, soil moisture (SM), pH, total nitrogen (TN), NO_3_^-^-N, NH_4_^+^-N were used as the indicators of soil properties; the abundance of total nematodes and trophic groups as the indicators of nematode communities. The analysis was performed with AMOS 7.0. Several tests were used to assess model fit, i.e. the χ^2^-test and its associated *P*-value, comparative fit index (CFI), goodness-of-fit (GFI) and root square mean error of approximation (RMSEM). Based on the results of goodness-of-fit tests, we excluded less predictive measures and the non-significant indicators and pathways, and retained the most informative variables. By stepwise removal of non-significant paths from the model, the final model that fit our data best was obtained.

## Results

### Soil properties

**Figure 1:**
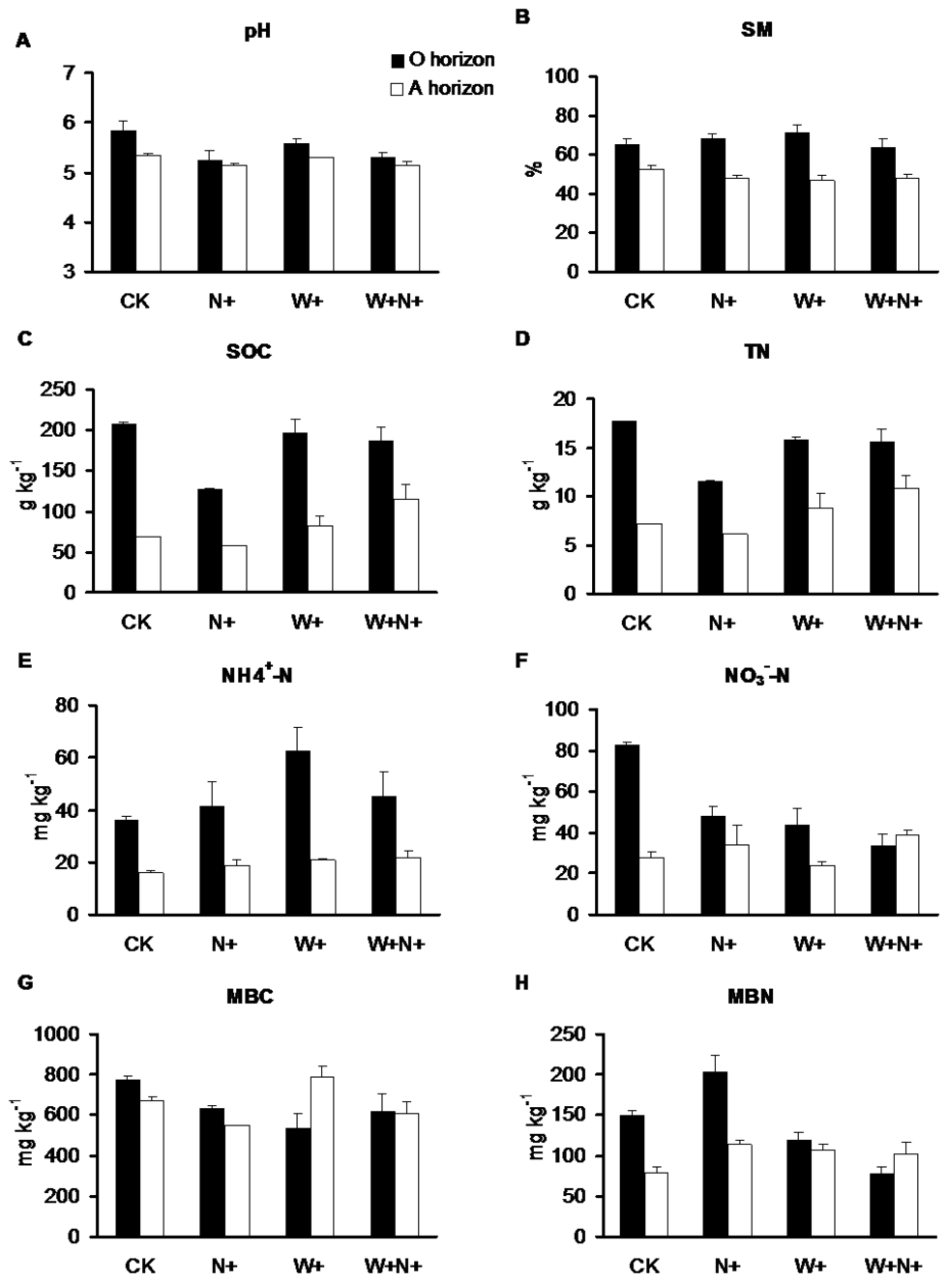
The effects of water addition, N addition and their interaction on soil properties. Bars indicate standard errors. SM, soil moisture; SOC, total soil organic carbon; TN, total nitrogen; MBC, microbial biomass carbon; and MBN, microbial biomass nitrogen.

In the O horizon, the values of SOC and NO_3_^-^-N were affected by water addition and N addition (*P* < 0.05) (Figure 1C, F). SOC was lower in the N+ treatment than in the other treatments (*P* < 0.01). NO_3_^-^-N were significantly decreased by W+ and W+N+ treatments compared to the control (*P* = 0.005 and 0.001, respectively). SM increased in the W+ treatment but this was not significant. 

In the A horizon, SOC and TN were significantly influenced only by water addition with higher values in W+N+ treatments than in the control (*P* < 0.05) (Figure 1 C, D). N addition effect on those of pH and MBC was found in both O and A horizons (*P* < 0.05). The values of pH were significantly lower in the N+ and W+N+ treatments than in the control (*P* < 0.05).

 The interactive effects of water and N addition were observed on TN (F = 12.88, *P* = 0.02), NH_4_^+^-N (F = 8.28, *P* = 0.045), MBC (F = 35.83, *P* = 0.004) and MBN (F = 33.03, *P* = 0.005) in the O horizon. The contents of MBC and MBN were significantly decreased by the W+N+ treatment compared to the control (*P* < 0.01).

### Soil nematode community structure and composition

**Figure 2:**
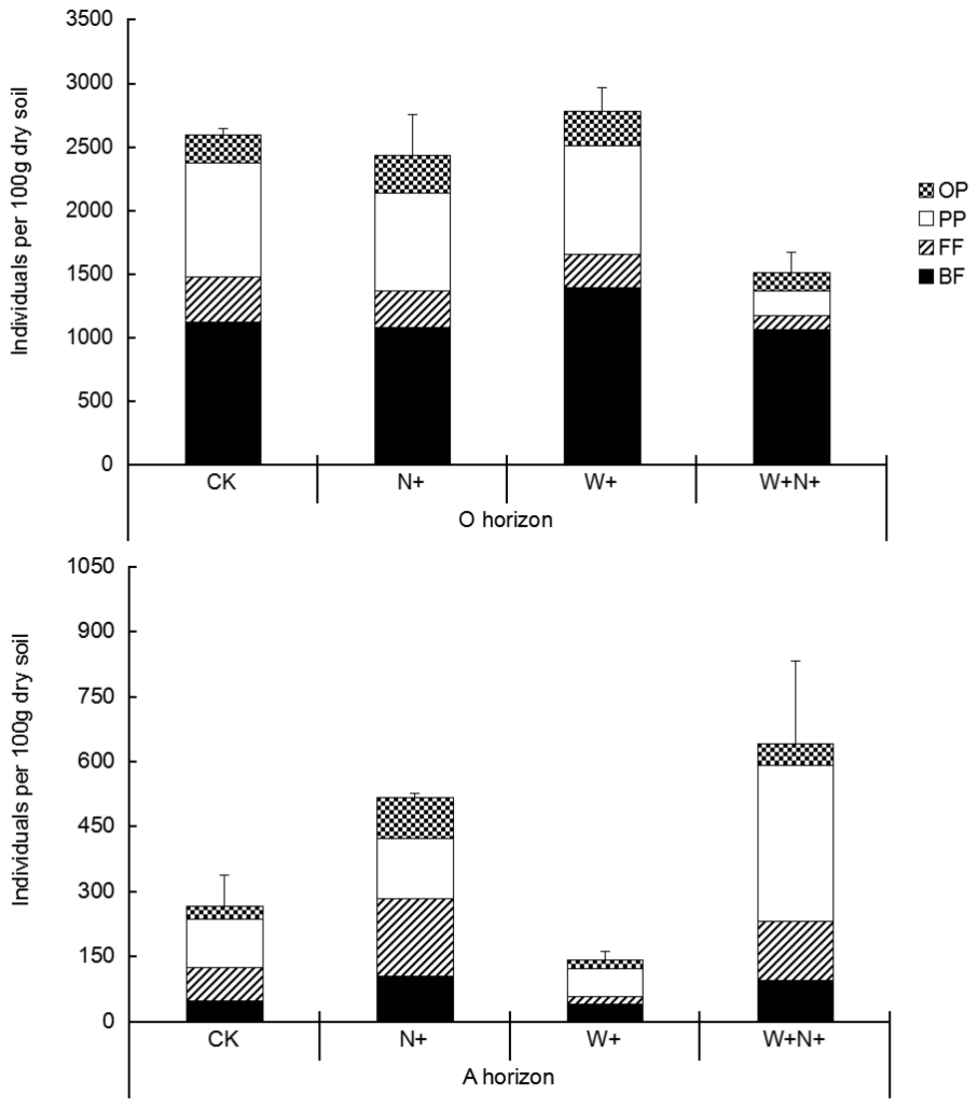
The abundances of total nematodes and trophic groups among different water or/and N addition treatments. Bars indicate standard errors. OP, omnivores-predators; PP, plant-parasites; FF, fungivores; BF, bacterivores.

 N addition influenced the total nematode abundance in both O (F = 11.37, *P* = 0.03) and A horizon (F = 15.02, *P* = 0.02), but water addition effect only in the O horizon (F = 8.32, *P* = 0.045). In the O horizon, the total nematode abundance was significantly lower in the W+N+ treatment than in other treatments (*P* < 0.05). In the A horizon, the total nematode abundance was higher in the W+N+ treatment than in the W+ treatment and the control (*P* < 0.05) (Figure 2).

 Among the four trophic groups, both water addition and N addition affected the abundances of fungivores and plant-parasites (*P* < 0.05). For omnivores and predators F = 53.08, *P* = 0.002) and for plant parasites (F = 8.84, *P* = 0.04), there was a significant interaction between the effects of water and N addition in the O horizon. The abundances of fungivores, plant-parasites and omnivores-predators were all significantly reduced by the W+N+ treatment compared to other treatments in the O horizon (*P* < 0.05) (Figure 2). In the A horizon, a significant effect on the abundance of fungivores of water addition and N addition was observed (*P* < 0.001) but the interaction between the two treatments was also significant (F = 19.22, *P* = 0.01).

 Forty-three nematode genera were identified in our study (Table 1), 17 of which were bacterivores, 5 fungivores, 11 plant parasites, and 10 omnivores-predators. The most abundant genera were *Helicotylenchus* and *Filenchus*. Water addition effects on bacterivores including *Wilsonema, Alaimus, Metateratocephalus, Eucephalobus* and *Plectus*, and the plant parasite *Malenchus* were significant in the O horizon while N addition influenced the bacterivore *Paramphidelus* in the O horizon (*P* < 0.05). 

**Table 1:** The relative abundance (%) of nematode genera among different treatments.

Genus	O horizon				A horizon			
	CK	N+	W+	W+N+	CK	N+	W+	W+N+
**Bacterivores**	34.8	44.7	48.8	60.9	16.8	17.5	23.6	22.6
*Achromadora*	0.7	2.3	1.9	2.6	0.0	1.0	0.7	0.8
*Alaimus*	1.3	1.0	2.0	3.3	0.6	2.5	1.7	2.6
*Paramphidelus*	1.3	2.3	0.7	2.6	1.2	0.5	1.0	0.3
*Bastiania*	2.7	2.3	1.0	3.0	1.9	1.1	1.0	1.1
*Acrobeloides*	0.3	0.0	0.0	0.7	0.0	0.0	0.0	0.0
*Cephalobus*	0.3	1.0	0.0	1.3	1.5	0.9	0.0	0.0
*Eucephalobus*	0.0	0.7	1.0	2.0	0.0	0.0	0.0	0.4
*Chronogaster*	0.0	0.7	2.3	1.0	0.6	0.0	1.1	2.2
*Odontolaimus*	0.7	1.3	1.3	1.0	0.6	1.5	0.8	2.2
*Anaplectus*	1.3	3.6	0.7	0.7	1.2	1.6	0.3	0.0
*Plectus*	3.6	2.3	7.2	7.5	4.6	2.0	7.6	5.3
*Wilsonema*	0.0	0.6	1.3	1.3	0.0	0.0	0.0	0.0
*Prismatolaimus*	10.6	7.1	9.2	5.9	1.8	3.0	5.7	4.8
*Pelodera*	0.7	0.7	2.3	4.6	0.0	0.7	1.8	0.3
*Rhabdolaimus*	0.3	3.9	2.6	6.2	2.7	1.0	0.0	0.0
*Metateratocephalus*	2.0	3.6	6.2	5.6	0.0	0.5	1.0	2.5
*Teratocephalus*	8.9	11.7	9.2	11.8	0.0	1.1	1.0	0.0
**Fungivores**	18.6	11.7	12.1	11.2	30.1	29.5	22.0	22.8
*Aphelenchoides*	1.7	0.7	2.3	2.3	3.4	0.0	0.0	0.0
*Diphtherophora*	1.0	2.6	4.2	0.3	2.5	1.9	2.5	2.4
*Dorylaimoides*	2.0	1.0	1.0	0.3	0.0	0.4	2.0	0.3
*Tylencholaimus*	4.3	3.2	2.0	2.0	6.5	8.2	1.3	0.0
*Filenchus*	9.6	4.3	2.6	6.2	17.8	19.0	16.2	20.1
**Plant-parasites**	34.4	31.6	30.2	18.7	42.9	30.7	42.7	46.6
*Macroposthonia*	2.3	2.9	1.3	0.7	7.0	3.3	5.1	0.3
*Nagelus*	2.0	0.0	1.3	0.0	2.5	0.0	2.9	0.3
*Tenunemellus*	1.3	1.3	1.3	0.7	0.0	0.4	0.0	1.9
*Helicotylenchus*	11.9	7.9	13.2	4.6	19.3	14.7	20.1	29.6
*Paratylenchus*	0.7	0.0	1.0	0.7	6.4	2.9	0.8	0.0
*Trichodorus*	2.7	2.6	0.3	0.7	0.0	1.8	1.3	0.3
*Basiria*	2.7	3.3	4.5	3.3	4.6	3.2	7.9	12.2
*Coslenchus*	0.0	0.3	1.0	1.6	0.0	0.0	1.7	0.0
*Lelenchus*	0.3	0.3	1.0	0.7	0.0	0.4	0.3	0.0
*Malenchus*	10.3	13.0	4.3	5.9	0.6	4.1	2.5	1.6
*Cephalenchus*	0.3	0.0	1.0	0.0	2.5	0.0	0.0	0.3
**Omnivores-predators**	12.3	12.0	8.9	9.2	10.2	22.4	11.7	8.0
*Aporcelaimellus*	0.7	0.0	0.0	0.7	0.6	2.7	0.0	0.0
*Clarkus*	0.0	0.7	0.0	0.0	1.5	0.7	1.1	0.0
*Mylonchulus*	0.7	0.3	1.7	0.3	0.6	0.7	0.3	0.4
*Epidorylaimus*	3.3	2.3	2.0	2.3	1.8	9.0	6.7	4.0
*Eudorylaimus*	0.7	1.6	0.3	0.3	0.6	0.9	1.0	0.0
*Microdorylaimus*	0.0	0.0	0.3	0.0	0.0	0.7	0.0	0.0
*Thonus*	2.0	1.3	0.0	0.7	0.6	2.6	0.3	1.9
*Mesodorylaimus*	0.0	0.3	0.7	0.0	0.0	0.4	1.0	0.0
*Tripyla*	3.7	5.5	3.3	4.9	3.1	4.0	1.3	0.7
*Trischistoma*	1.3	0.0	0.6	0.0	1.2	0.7	0.0	1.0

 In the O horizon, both the water addition and N addition effects on TD (*P* = 0.004 and 0.027, respectively) and NCR were significant (*P* = 0.002 and 0.010, respectively); TD was significantly decreased by the W+ (*P* = 0.025) and W+N+ treatments (*P* < 0.001) compared to the control, and NCR was lower in the control than in other treatments (*P* < 0.05) (Table 2). In the A horizon, water addition, N addition and their interactive effects were all found to be significant on λ (P = 0.001, 0.012 and 0.007, respectively); the W+N+ treatment significantly increased λ compared to other treatments (*P* < 0.01).

**Table 2:** Nematode ecological indices among different N and water addition treatments.

O	TD	λ	H'	MI	NCR	CI
CK	2.97±0.09	0.08±0.00	2.55±0.05	2.91±0.02	0.75±0.01	100.00±0.00
N+	2.86±0.11	0.08±0.01	2.71±0.05	2.99±0.03	0.80±0.01	82.22±17.78
W+	2.54±0.16	0.11±0.02	2.61±0.17	2.86±0.08	0.84±0.00	36.36±5.25
W+N+	1.91±0.07	0.06±0.00	2.84±0.02	2.75±0.04	0.91±0.02	17.46±3.67
W	**	ns	ns	ns	**	**
N	*	ns	ns	ns	*	Ns
W×N	ns	ns	ns	ns	ns	Ns
A						
CK	3.15±0.27	0.11±0.02	2.28±0.22	2.72±0.22	0.36±0.05	100.00±0.00
N+	3.60±0.06	0.09±0.01	2.46±0.05	3.07±0.03	0.36±0.06	87.50±7.22
W+	3.17±0.14	0.12±0.02	2.14±0.10	2.95±0.15	0.68±0.05	60.00±23.09
W+N+	2.44±0.12	0.29±0.01	1.65±0.02	2.52±0.13	0.38±0.10	85.71±8.25
W	*	**	*	ns	*	Ns
N	ns	*	ns	ns	ns	Ns
W×N	*	**	ns	*	ns	Ns

Notes: * and ** indicate significant differences at *P* < 0.05 and *P* < 0.01, respectively; ns indicates no significant difference. O, organic horizon; A, mineral horizon; TD, trophic diversity; λ, dominance; H', diversity index; MI, maturity index; NCR, the nematode channel ratio; CI, channel index.

### Structural equation modeling (SEM) analysis

**Figure 3:**
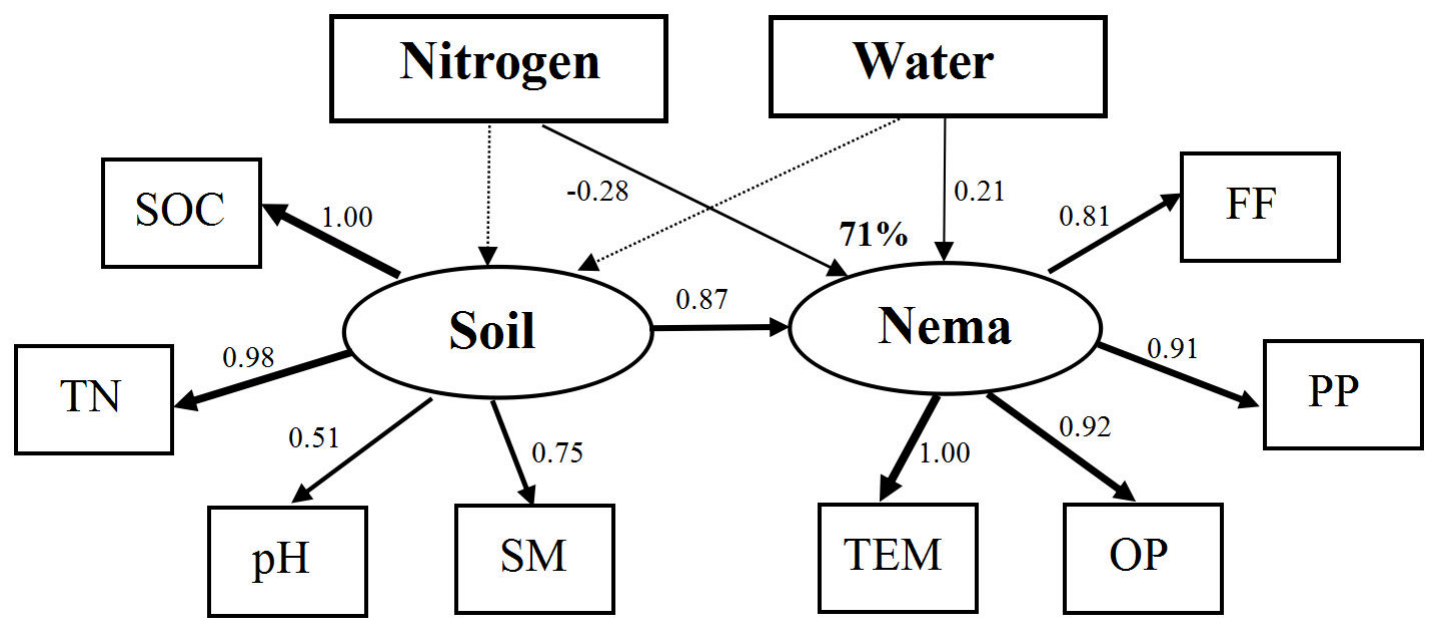
Structural equation models of water addition and N addition effects on soil nematode communities (χ^2^ = 41.08; df = 30; *P* = 0.09; GFI = 0.79; CFI = 0.96; RMSEA = 0.13). Numbers on arrows are standardized path coefficients. Width of the arrows indicates the strength of the causal influence. Dashed and straight lines represent significant and non-significant pathways, respectively. Nitrogen, N addition effect; Water, water addition effect; Nema, soil nematode communities; Soil, soil properties; SOC, total soil organic carbon; TN, total nitrogen; SM, soil moisture; TEM, total nematodes; FF, the abundance of fungivores; PP, the abundance of plant-parasites; and OP, the abundance of omnivores-predators.

 Soil nematode community responses to water or N addition treatments were analyzed by SEM model regardless of soil horizons (Table 3). The final models adequately fit the data on the effect of water and nitrogen addition on soil nematode communities (χ^2^ = 41.08; df = 30; *P* = 0.09; GFI = 0.79; CFI = 0.96; RMSEA = 0.13; standardized path coefficients are given in Figure 3). The SEM analysis indicates that water addition had the positive and N addition a negative effect on soil nematode composition. The model explained 71% of the variance in nematode communities. The effects of N and water addition on soil properties were not significant. The final SEM models indicated that soil nematode communities were more directly influenced by water and N addition.

**Table 3:** The SEM model results showing the estimates, standard errors (SE), critical ratio (CR) and the P value.

Pathway	Estimate	SE	CR	P
Water → Soil	-29.42	23.38	-1.26	0.21
Water → Nema	0.44	0.21	2.06	0.04
Nitrogen → Soil	17.04	23.38	0.73	0.47
Nitrogen → Nema	-0.59	0.20	-2.99	0.00
Soil → Nema	0.02	0.00	6.33	0.00
Soil → TC	1.00			
Soil → TN	0.07	0.00	25.62	0.00
Soil → SM	0.00	0.00	5.49	0.00
Soil → pH	0.00	0.00	3.48	0.00
Nema → FF	0.68	0.10	6.82	0.00
Nema → PP	0.85	0.11	7.87	0.00
Nema → OP	1.00			
Nema → TEM	1.04	0.09	11.69	0.00

Estimate indicate estimate of regression weight.

## Discussion

### The effects of water and N addition on soil properties

In our study, soil pH was significantly decreased by the N addition effect. Numerous studies have shown that atmospheric N inputs lead to soil acidification [5,52–54]. Soil acidification following N addition has been proposed as the important factor affecting soil nematodes [55,56].

Effects of N inputs on soil C cycling processes have been documented in temperate forest ecosystems [5,57]. In our study, the content of SOC was decreased in the N addition treatment in the O horizon. The accumulations of soil organic matter or organic carbon mainly result from plant litter decomposition. However, other studies have found that N addition could reduce the abundance and biomass of microbes and soil fauna as primary decomposers of plant litter [54,58]. The low litter composition rates due to N addition were the possible reason for the reduction of soil carbon content. 

Changes in precipitation influence soil moisture status and will profoundly impact soil biochemical properties [15,59,60]. In our study, the contents of NO_3_^-^-N were significantly decreased by water addition effect in the O horizon. NO_3_^-^-N not taken up by plants was leached downward to subsoil by rainfall and irrigation water, or flowed into groundwater by runoff [61]. The leaching of NO_3_^-^-N from the upper soil layer was at least partly hydrologically driven and preferentially flowed through soil macropores, and then precipitation promoted its accumulation in the deeper layer [57,62,63]. The loss of NO_3_^-^-N was also related to the climatic and soil conditions in this area. Xu et al. (2009) showed that the N addition increased the accumulation of NO_3_^-^-N in soil solutions at 15 cm and 60 cm depths in Changbai Mountain and that the concentrations of inorganic N differed significantly among sampling dates [54]. In the low pH soil environment, the diffusion of NO_3_^-^-N without specific adsorption property will even be stimulated further with rainfall [61].

### The effects of water addition and N addition on soil nematode communities

 In our study, N addition decreased the soil nematode communities in the O horizon, which showed a decreasing trend in total nematode abundance and trophic diversity. The results are in line with other studies [18,55], which also showed that N addition generally decreased total abundance and diversity of nematodes. However, the opposite trend was found in the A horizon, which suggest that some nematodes could survive by migrating to subsoil in response to the N addition. Some studies found that soil nematodes are highly sensitive to precipitation and that nematode abundance increases with increasing precipitation in forest ecosystems [1,15]. In our study, the total nematode abundance also increased in the water addition treatment, but the trend changed after the N addition.

We also observed that nematode trophic groups except for bacterivores showed similar responses to the N addition. Fungivores and plant-parasites were decreased by N addition in the O horizon. According to a study on the effect of N inputs on organic matter decomposition, N additions have a direct toxic effect on some saprophytic fungi by inhibiting their enzymes [64], and this can affect soil nematodes indirectly. N addition strongly reduced soil fungi, and as a consequence of food resource reduction [65], the fungivores were likely decreased, which may be the reason for the decreasing abundance of fungivores observed. In our study, the abundance of plant parasites was higher and the content of NH_4_^+^-N was lower in the control than in other treatments. Wei et al. (2012) showed that the nematicide toxicity produced by the increasing ammonium concentration following the N addition might explain the reduction in plant parasites [18]. The interactive effect of water and N addition significantly decreased the abundance of omnivores-predators. This is because omnivores-predators as *K*-strategists are sensitive to the environmental change and apt to be affected by nutrient addition [66]. 

In terms of nematode ecological indices, the significant water and N addition effects on TD and NCR were mostly explained by the variation in the abundances of fungivores and plant-parasites. These results indicate that N deposition and precipitation could affect nematode community through changes in the trophic group level.

### The interactive effects of water addition and N addition on soil nematode communities

 A lower total nematode abundance in the O horizon was observed in the W+N+ treatment. The N addition effect on soil nematode communities was significantly affected by water addition. Water availability influenced community sensitivity to N, and therefore aggravated or intensified the declining trend of the soil nematodes following N addition [14,67]. It has been showed that atmospheric N inputs, N mineralization or nitrate leaching were all directly linked to precipitation [11,68]. The effects of N addition on soil nematode communities depended on water addition in our study. The synergistic effects of precipitation and N deposition as the driving force of nematode community existed and might be more complex and stronger than each effect alone. 

### Direct and indirect effect of N and water addition on soil nematode communities

 SEM analysis revealed that soil nematode communities responded directly to N and water addition. The N addition effect was more obvious than the effect of water addition. Soil properties including SOC, TN, pH and SM all affected nematode communities, especially total nematode abundance, omnivores-predators, plant-parasites and fungivores. In our study, SEM results revealed that the direct effects of N addition on soil nematode communities were more important than the indirect effects through alterations in soil abiotic properties. Similar conclusions were also attained in the studies of Wei et al. (2012) and Li et al. (2013) on the N addition on nematode communities [18,69]. Although the significant effects of water and N addition on soil properties were not shown in the SEM model, the responses of soil nematodes were highly sensitive to their effect. This suggests that the minimal variations in soil properties lead to the clear changes in soil nematode communities. This study further suggests that soil nematodes are sensitive indicators to environments as they exhibited rapid responses to N deposition and precipitation. In summary, our results provide strong evidence that soil nematodes as bioindicators were highly sensitive to the climate changes, which is of great importance to clarify the feedback between climate change and terrestrial ecosystems.
